# 

**Published:** 1881-04

**Authors:** 


Vol. I. APRIL, 1881. No. 4.

### THE

### HOMŒOPATHIC PHYSICIAN:

A MONTHLY JOURNAL OF MEDICAL SCIENCE.

*"Magna est veritas et praevalebit."*

E. J. LEE, M. D.
EDITOR.

